# Effect of reduced foot and ankle sensation on postural response to hip abductor/foot everter vibration

**DOI:** 10.1186/1757-1146-8-S1-A4

**Published:** 2015-04-20

**Authors:** Sam Glasser, Joanne Paton, Richard Collings, Jonathan Marsden

**Affiliations:** 1 BEUP, School of Health Professions, Faculty of Health and Human Sciences, Plymouth University, Plymouth, UK; 2 BEUP, Torbay and Southern Devon Health and Care NHS Trust, Devon, UK

## Aims

The aim of this study was to assess whether postural responses induced by vibratory perturbations of the hip abductors and foot everters were modified when foot/ankle sensation was experimentally reduced.

## Background

Successful integration of vestibular, visual and somatosensory information results in motor responses to maintain upright stance. When one or more of these senses have reduced efficacy, balance can be affected.

When proprioception is reduced, people show greater postural sway amplitudes, resulting in increased centre of pressure excursions as seen in diabetic peripheral neuropathy. This is the primary cause of postural instability in the diabetic population. To compensate for this, re-weighting between sensory modalities can occur. Reduced peripheral sensation, for example, can result in an increased gain of the postural response to galvanic vestibular stimulation. Particularly in people with distal sensory loss, balance may depend on the ability to effectively reweight remaining information from within the somatosensory system.

## Methods

Sixteen healthy subjects were investigated (9 female, 7 male age 40±15yrs) pre and post foot/ankle cooling. Cooling provided a method of reducing foot and ankle sensation whereby replicating to a degree, peripheral neuropathy. Subjects stood with their eyes closed whilst a 2 s vibratory stimulus was applied to the left or right hip abductor or foot everter to perturb balance. The postural responses to these perturbations were measured at the knee, pelvis, trunk and head using a 3D motion analysis system (Codamotion, Leicestershire). Medio-lateral ground reaction forces and centre of pressure motion were simultaneously recorded via a force plate (Kistler, UK).

## Results

Postural responses to hip and ankle vibration, pre and post cooling were analysed using repeated measures ANOVA.

In response to ankle vibration the pelvis translated and tilted toward the side of stimulation. In response to hip vibration the pelvis translated and tilted away from the side of stimulus. Post cooling the direction of the response to ankle vibration remained unchanged however there was a reduction in the amplitude of pelvic tilt (F(6.2)=P<0.05). Post cooling the direction of the response to hip vibration also remained unchanged however there was an increase in the amplitude of pelvic tilt (F(5.2)=P<0.05).

## Discussion

By experimentally reducing foot/ankle sensation through cooling there was a reduction in the amplitude of pelvic tilt in response to ankle vibration and increased amplitude of pelvic tilt in response to hip vibration. This study suggests that in the presence of distal sensory loss the body continues to maintain postural stability by reweighting more proximal sensory inputs- a possible advantage for those with peripheral neuropathies. However in the presence of peripheral neuropathy the ability to use these more proximal senses may be dependent on flexibility/strength in these proximal segments. Range of motion on other joints may also play an important role in providing additional sensory positional feedback to facilitate the increased gain of hip proprioceptive and vestibular postural responses.

Developing our knowledge in frontal plane movement and medio-lateral stability informs future research aimed at developing a targeted program for balance enhancement in those with diabetic peripheral neuropathy.

